# The underlying molecular mechanism and identification of transcription factor markers for laryngeal squamous cell carcinoma

**DOI:** 10.1080/21655979.2020.1862527

**Published:** 2021-01-04

**Authors:** Bin-Yu Mo, Guo-Sheng Li, Su-Ning Huang, Wei-Ying He, Li-Yuan Xie, Zhu-Xin Wei, Ya-Si Su, Yue Liang, Li Yang, Cheng Ye, Wen-Bin Dai, Lin Ruan

**Affiliations:** a Department of Otolaryngology, Liuzhou People’s Hospital of Guangxi, Liuzhou, Guangxi Zhuang Autonomous Region, P.R. China; b Department of Radiotherapy, First Affiliated Hospital of Guangxi Medical University, Nanning, Guangxi Zhuang Autonomous Region, P.R. China; c Department of Radiotherapy, Guangxi Medical University Cancer Hospital, Nanning, Guangxi Zhuang Autonomous Region, P.R. China; d Department of Pathology, Liuzhou People’s Hospital, Liuzhou, Guangxi Zhuang Autonomous Region, P.R. China

**Keywords:** Laryngeal squamous cell carcinoma, transcription factor, homeobox B13, RNA sequencing, prognosis

## Abstract

The screening and treatment of laryngeal squamous cell carcinoma (LSCC) still perplexes clinicians, making it necessary to explore new markers. To this end, this research examined the underlying molecular mechanism of LSCC based on high-throughput datasets (*n* = 249) from multiple databases. It also identified transcription factors (TFs) independently associated with LSCC prognosis. Through Gene Ontology and Kyoto Encyclopedia of Genes and Genomes analyses, differential expression genes of LSCC were deemed relevant to the extracellular matrix and its related structures or pathways, suggesting that the extracellular matrix plays an important role in LSCC. At the same time, several hub genes that may also have important roles in LSCC were identified via protein–protein interaction analysis, including *CDC45, TPX2, AURKA, KIF2C, NUF, MUC1, MUC7, MUC4, MUC15,* and *MUC21*. Eight unreported LSCC prognostic TFs – *BCAT1, CHD4, FOXA2, GATA6, HNF1A, HOXB13, MAFF,* and *TCF4* – were screened via Kaplan–Meier curves. Cox analysis determined for the first time that *HOXB13* expression and gender were independently associated with LSCC prognosis. Compared to control tissues, elevated expression of *HOXB13* was found in LSCC tissues (standardized mean difference = 0.44, 95% confidence interval [0.13–0.76]). *HOXB13* expression also makes it feasible to screen LSCC from non-LSCC (area under the curve = 0.77), and *HOXB13* may play an essential role in LSCC by regulating *HOXB7*. In conclusion, *HOXB13* may be a novel marker for LSCC clinical screening and treatment.

## Introduction

Laryngeal carcinoma (LC) accounts for approximately 27% of head and neck cancer in the world [1], and its main pathological subtype is laryngeal squamous cell carcinoma (LSCC). In China, there were 26,400 new cases of LC and 14,500 related deaths in 2015 [2]. In 2018, the number of newly confirmed global cases of LC was more than 177,000, with more than 90,000 related deaths [1]. In recent years, improvements in LC morbidity and mortality have not inspired optimism. Taking the United States as an example, from 1975 to 2011, the five-year survival rate for those with LC decreased from 66% to 63%, and, based on data from 2012, it is predicted that new incidences of and deaths from LC will have risen slightly again in 2020 [3,4].

At present, clinical strategies for treating LC include surgery, radiotherapy, chemotherapy, and systemic treatment [5–8]. Early treatment has achieved good results with the 5-year disease-specific survival >75% [9–11]. For patients with advanced LC, total laryngectomy may be the only option [5,12]; however, problems caused by this surgery (such as loss of speech and swallowing dysfunction) greatly reduce patient quality of life. Even worse, early LC screening is difficult due to the lack of obvious specific symptoms in most cases, which is conducive to tumor growth and spread [13]. More than 70% of LC patients are diagnosed at stage III or IV [14,15], presenting further severe treatment challenges.

Current studies concerning LSCC pathogenesis have indicated that its occurrence and development may be related to drinking [16], smoking [15], asbestos exposure [17], and human papillomavirus (HPV) infection [18]. Other research has shown that LSCC pathogenesis may be related to multiple molecules, such as *Shp2* [19] and *HPV16* [18], and various forms of long noncoding RNA and microRNA [18,20]. The latter involve signaling pathway changes, such as *Ras*/*Raf*/*Mek*/*Erk* [21], *PTEN*/*Akt* [22], and *PI3K*/*Akt* [23]. These indicate the complexity of LSCC pathogenesis, and the molecular pathological mechanisms of its occurrence and development are far from fully elucidated. Therefore, further study is needed to determine LSCC’s molecular pathological mechanism and explore new markers suitable for early clinical screening and treatment.

Transcription factors (TFs) help initiate eukaryote transcription and can regulate gene expression by interacting with cis-acting elements. Thus, they play an important role in tumorigenesis and development. Previous studies have shown multiple TFs associated with tumor prognosis. For example, *SIX1* can promote the growth of breast cancer by enhancing the expression of multiple glycolytic genes; it is also considered a good predictor of breast cancer prognosis [24]. *GATA4* can promote the aging of lung cancer cells, and reduced *GATA4* expression levels are associated with poor lung cancer prognosis [25]. In this vein, studies concerning LSCC and TF have suggested that *SOX2* overexpression may indicate poor patient prognosis [26]; *ATF-3* [27] and Forkhead Box M1 [28] are also closely related to LSCC prognosis. The potential importance of TFs in LSCC development makes exploring TFs related to LSCC prognosis (PRTFs) essential for elucidating the molecular pathological mechanism of LSCC and exploring new markers for screening and treatment of the disease.

Presently, high-throughput sequencing technologies (gene chips, RNA-Seq, etc.) are widely used in the biomedicine field. In this research, we collected high-throughput datasets from ArrayExpress (https://www.ebi.ac.uk/arrayexpress/), Gene Expression Omnibus (GEO, https://www.ncbi.nlm.nih.gov/geo/), Oncomine (https://www.oncomine.org/resource/login.html), and the Cancer Genome Atlas (TCGA). Based on analyses of these datasets and published reports by scholars, we explored the potential molecular mechanism of LSCC. Moreover, from the TF level, we identified new markers that may be suitable for clinical LSCC screening and treatment.

## Materials and methods

### Collection of gene chip data

LSCC-related datasets were retrieved and screened from the ArrayExpress, GEO, Oncomine, and TCGA. The overall retrieval strategy set to: ‘(laryn * OR glotti * OR (vocal muscle)) AND (mRNA OR gene).’

The inclusion criteria were as follows [1]: the research species was *Homo sapiens* [2]; the samples were taken from human larynx tissue [3]; the samples were pathologically diagnosed as LSCC; and [4] the dataset contained at least three of both LSCC and control samples. The exclusion criteria were as follows [1]: the study contained no cancer or control samples; and [2] the data were duplicated or incomplete.

### Collection of RNA-Seq data and corresponding sample clinical information

The RNA-Seq dataset of TCGA was included and downloaded from the Genomic Data Commons Data Portal (https://portal.gdc.cancer.gov/repository). Clinical information about corresponding samples in the dataset was collected from the University of California, Santa Cruz, Xena database (http://xena.ucsc.edu/).

### Underlying molecular mechanism of LSCC

Gene chip data were log_2_ (x + 1) log-transformed to screen for differential expression genes (DEGs). For RNA-Seq, raw count data was prepared for DEGs screening, while log_2_ (x + 1) log-transformed data were prepared to screen for and identify prognostic TFs. DEGs were screened by using the limma [29] and edgeR [30] packages of R (v3.6.1). The screening conditions were ‘| log2 (fold change) | ≥1,’ adjusted *p* value <0.05. The frequency of occurrence for upregulated-DEGs (up-DEGs) and downregulated-DEGs (down-DEGs) in all datasets was counted and sorted to select high-frequency DEGs in all datasets.

Gene Ontology (GO) analysis [31] and Kyoto Encyclopedia of Genes and Genomes (KEGG) pathway enrichment analysis [32] were conducted via the clusterProfiler package [33] in R for the identified up-DEGs and down-DEGs, respectively. The visualization of GO and KEGG analyses were performed with the GOplot package [34] in R. The protein–protein interaction (PPI) analysis for up-DEGs and down-DEGs was performed in the Search Tool for the Retrieval of Interacting Genes/Proteins database (https://string-db.org/cgi/input.pl?sessionId=Ya5dxqsyKmY7&input_page_show_search=off). Hub genes in the up-DEGs and down-DEGs were screened according to the Maximal Clique Centrality algorithm of the CytoHub plug-in in Cytoscape (v3.7.2) [35].

### Identification of TF markers for screening and treatment of LSCC

Binding Analysis for Regulation of Transcription (BART, https://faculty.virginia.edu/zanglab/bart/) contains more than 6,000 chromatin immunoprecipitation sequencing (ChIP-Seq) data. As a bioinformatics analysis tool, BART can be utilized to predict TFs of genes based on ChIP-Seq data [36]. Through BART, we explored TFs of the up-DEGs with a *p* < 0.01, while these TFs were identified as upregulated-TFs (up-TFs). Similarly, downregulated-TFs (down-TFs) based on the down-DEGs were also selected.

Based on the median expression level of TFs, LSCC samples in the RNA-Seq dataset were divided into high-expression and low-expression groups. With these groups, the Kaplan–Meier curve, univariate Cox analysis, and multivariate Cox analysis were performed to identify LSCC PRTFs and TFs independently related to prognosis (PIRTFs). Forest plots with standardized mean difference (SMD) were applied to evaluate the expression of PIRTFs in LSCC and non-LSCC samples. Funnel plots with Egger’s test were utilized to evaluate publication bias of SMD results. The area under the curve (AUC) of the summary receiver-operating curve (sROC) was used to evaluate the screening effect of PIRTFs in distinguishing LSCC from non-LSCC samples. Forest plots, funnel plots, and sROC were drawn in Stata 15.

### Prediction of potential PIRTF targets

Primary PIRTF targets were drawn from Cistrome Data Browser [37] with scores of not less than two. Secondary PIRTF targets were based on the intersection of primary targets, up-DEGs of LSCC, and positively co-expressed genes (PCEGs, Pearson coefficient ≥0.3, *p* < 0.05) of PIRTFs. The JASPAR database [38,39] and MEME Suite online tool [40] were utilized to explore matched sequences between PIRTF motifs and promoter regions of secondary targets. Through secondary targets, potential PIRTF targets were screened via ChIP-Seq data in Cistrome Data Browser [37].

### Statistical analysis

A 95% confidence interval (CI) of SMD that does not contain zero indicates that the SMD result is statistically significant. An Egger’s test *p* value of greater than 0.1 indicates no publication bias of SMD results. The range of AUC is 0 to 1, and the closer the AUC is to 1, the greater the effect of PIRTFs in screening LSCC. Unless otherwise specified, a *p* value lower than 0.05 in this study indicates statistically significant difference.

**Figure 1.**
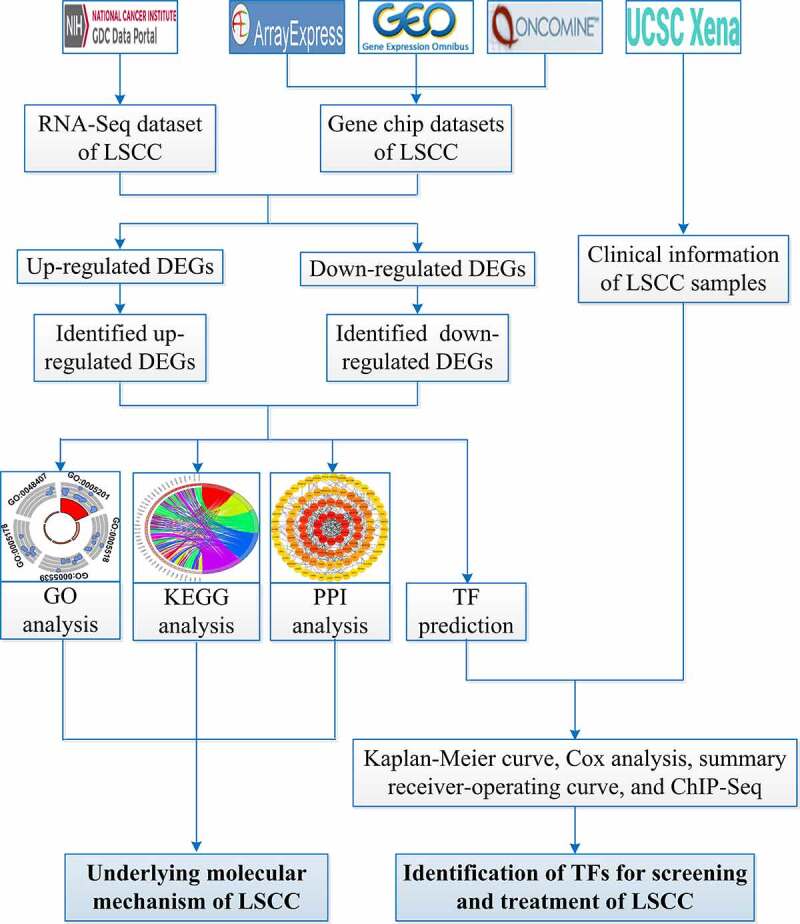
Flow chart of this study. LSCC: laryngeal squamous cell carcinoma; GO: Gene Ontology; KEGG: Kyoto Encyclopedia of Genes and Genomes; PPI: protein-protein interaction; DEGs: differential expression genes; TF: transcription factor; ChIP-Seq, chromatin immunoprecipitation sequencing

**Figure 2.**
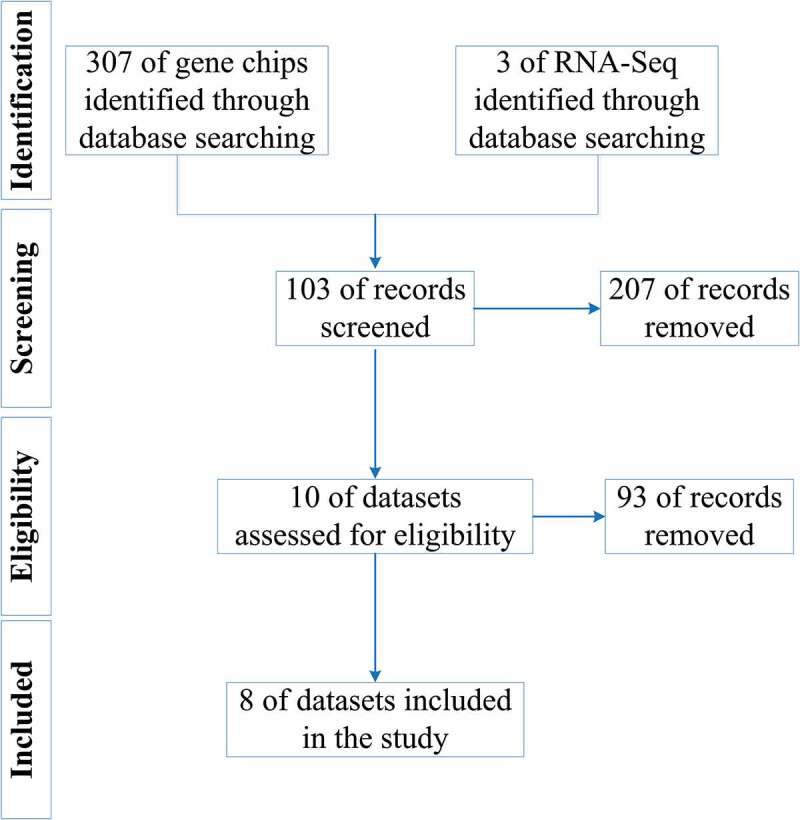
Screening processes for datasets included in the study

Figure 1 shows the overall study design. The processes for selecting the gene chips and RNA-Seq datasets are illustrated in Figure 2.

## Results

### Collection of gene chip data

As of 30 March 2020, seven gene chip datasets (E-MEXP-44, GSE29330, GSE51985, GSE58911, GSE59102, GSE84957, and GSE107591) were included in the research, containing 70 LSCC samples and 56 non-LSCC control samples. The basic information for each gene chip is shown in Table 1.

**Table 1.** Basic information of each dataset

Dataset	Database	Platform	*n* ^a^ of samples		*n* of DEGs ^b^	
			LSCC ^c^	Non-LSCC	Up-DEGs ^d^	Down-DEGs ^e^
E-MEXP-44	ArrayExpress	A-AFFY-1/ A-AFFY-32	8	8	66	59
GSE29330	GEO ^f^	GPL570	3	5	436	303
GSE51985	GEO	GPL10558	10	10	382	458
GSE58911	GEO	GPL6244	7	7	75	284
GSE59102	GEO	GPL6480	29	13	1493	1667
GSE84957	GEO	GPL17843	9	9	618	422
GSE107591	GEO	GPL6244	4	4	16	15
RNA-Seq	TCGA ^g^	-	111	12	5557	2940

^a^: number; ^b^: differential expression genes; ^c^: laryngeal squamous cell carcinoma; ^d^:up-regulated differential expression genes; ^e^:down-regulated differential expression genes; ^f^:Gene Expression Omnibus; ^g^:the Cancer Genome Atlas.

### Collection of RNA-Seq data and corresponding sample clinical information

The RNA-Seq data, containing 123 samples – 111 LSCC samples and 12 non-LSCC control samples – and clinical information for these samples, were downloaded from the Xena database; basic information about the RNA-Seq dataset is shown in Table 1.

### DEGs identification

**Figure 3.**
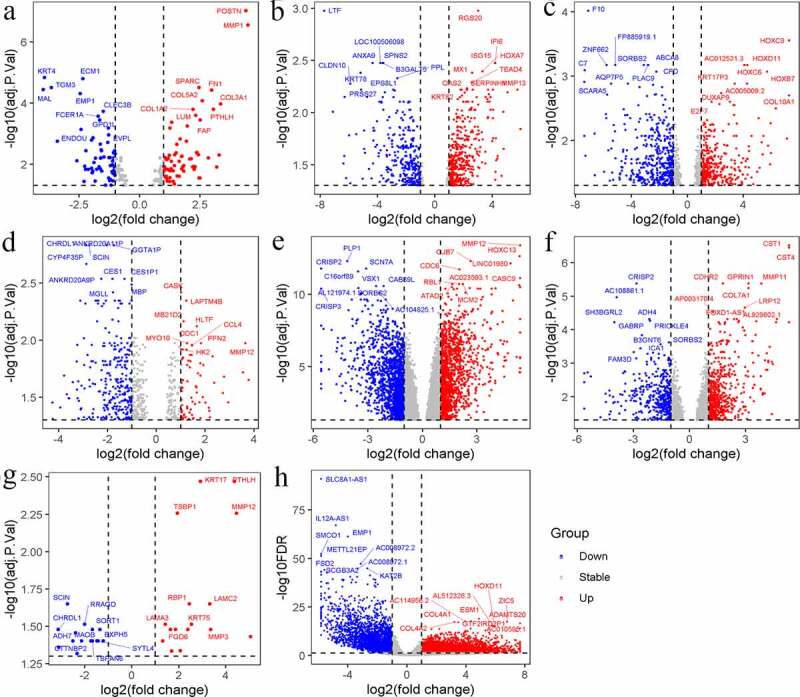
Volcano plots of differential expression genes in E-MEXP-44 (a), GSE29330 (b), GSE51985 (c), GSE58911 (d), GSE59102 (e), GSE84957 (f), GSE107591 (g) and TCGA (h). Up: up-regulated genes; Down: down-regulated genes

The up-DEGs and down-DEGs of each dataset were screened (Table 1, Figure 3). The researchers excluded genes with inconsistent expression differences between datasets – that is, genes whose expressions were upregulated in one dataset and downregulated in another. To be categorized as an identified up-DEG or down-DEG, genes were required to have been found in no fewer than three of the eight datasets. Using this metric, 458 identified up-DEGs and 493 identified down-DEGs were selected.

### GO analysis

**Figure 4.**
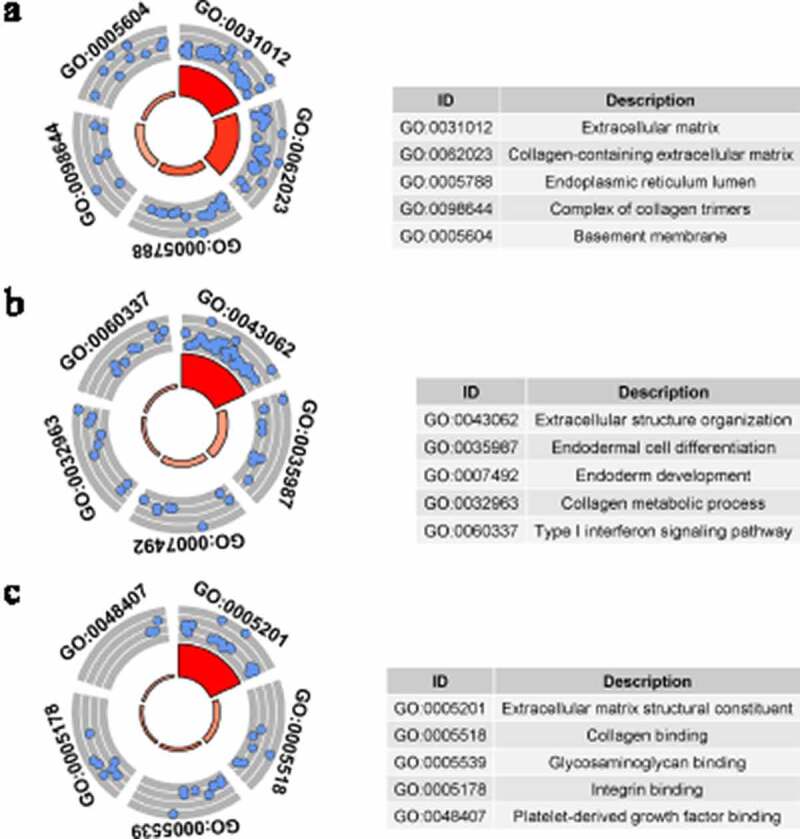
Gene Ontology (GO) analysis for identified up-regulated differential expression genes. Cellular components (a), biological processes (b) and molecular functions (c) of identified up-regulated differential expression genes. The blue nodes in the concentric circles represent genes clustered in specific GO terms. The larger size and darker color of the internal departments represent the more significant enrichment of GO term

**Figure 5.**
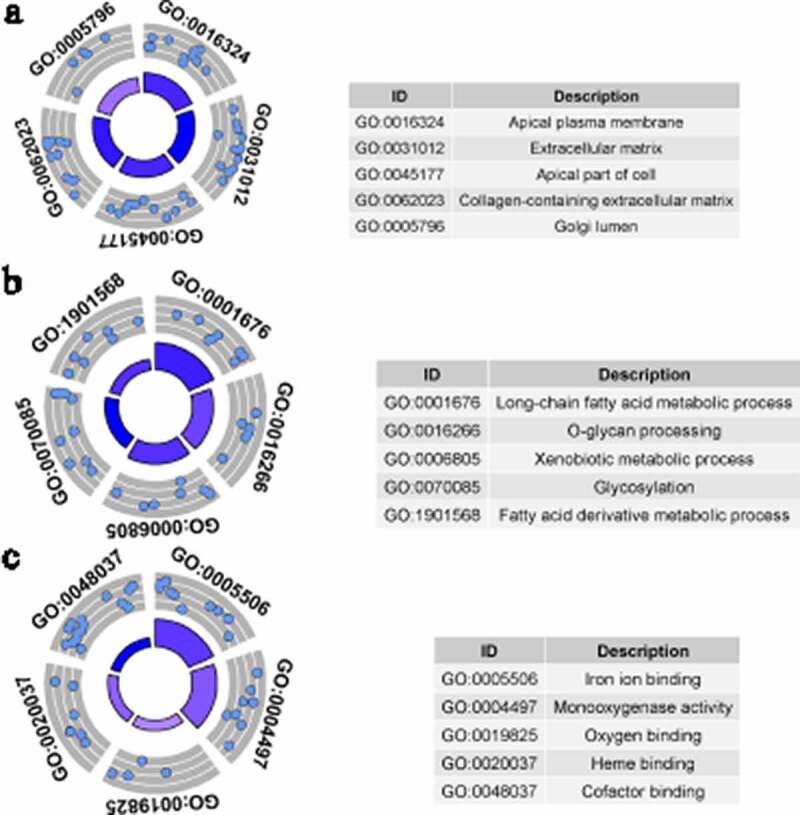
Gene Ontology (GO) analysis for identified down-regulated differential expression genes. Cellular components (a), biological processes (b) and molecular functions (c) of identified down-regulated differential expression genes. The blue nodes in the concentric circles represent genes clustered in specific GO terms. The larger size and darker color of the internal departments represent the more significant enrichment of GO term

GO analysis was conducted by studying three types of GO terms: cellular components (CC), biological processes (BP), and molecular functions (MF). GO terms with *p* < 0.05, and at least five enriched genes, were considered statistically significant. Figures 4 and 5 display the five most enriched GO terms based on analysis of identified up-DEGs and down-DEGs. Based on identified up-DEGs, the top-enriched GO term of CC was ‘extracellular matrix,’ while those of BP and MF were ‘extracellular structure organization’ and ‘extracellular matrix structural constituent,’ respectively. For identified down-DEGs, the three top-enriched GO terms were ‘apical plasma membrane’ (CC), ‘long-chain fatty acid metabolic process’ (BP), and ‘iron ion binding’ (MF).

### KEGG pathway enrichment analysis

**Figure 6.**
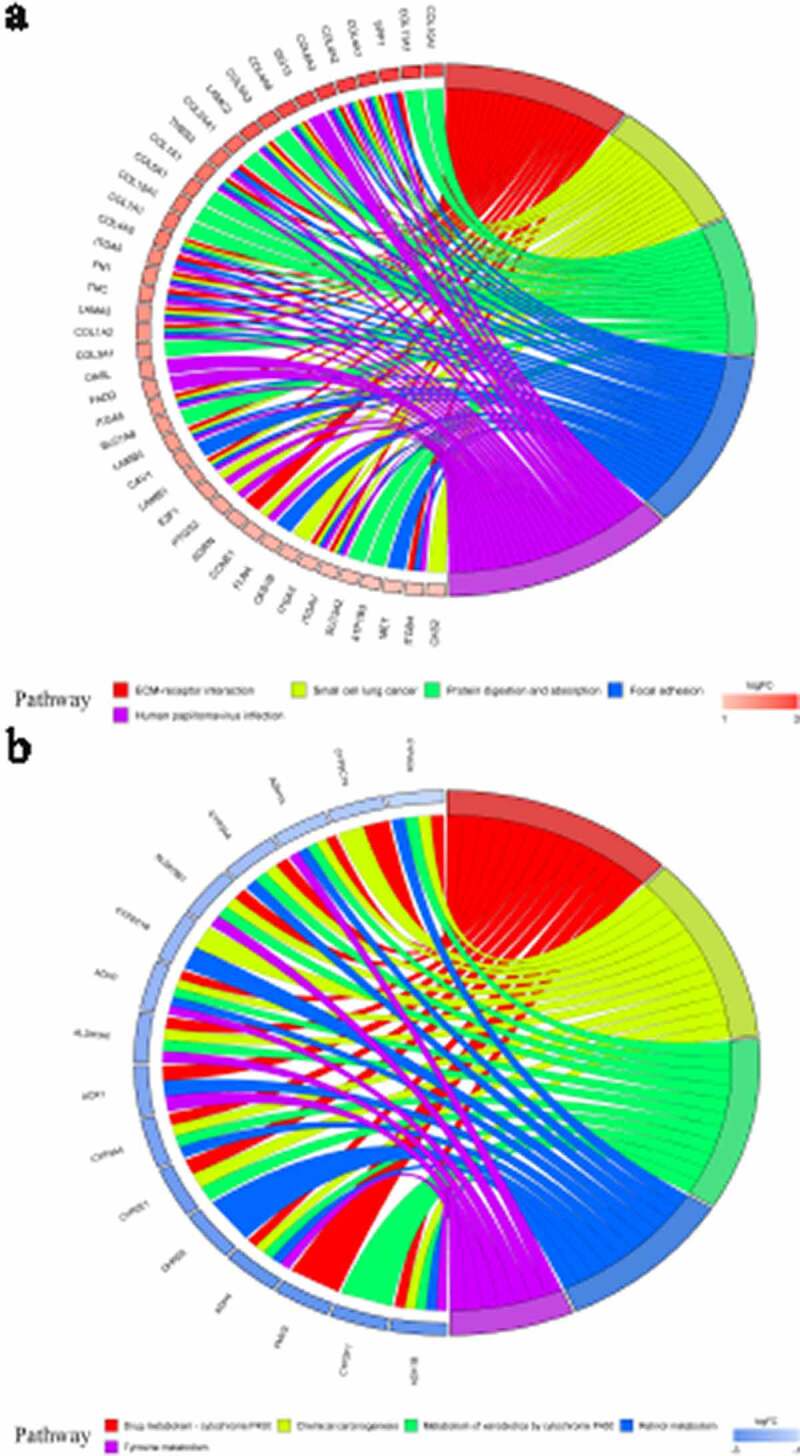
Kyoto Encyclopedia of Genes and Genomes (KEGG) analysis of identified up-regulated differential expression genes (a) and identified down-regulated differential expression genes (b). Different color bands correspond to different KEGG enrichment pathways

Through KEGG pathway enrichment analysis, quite a few enriched signaling pathways were related to tumorigenesis and tumor development (Figure 6). For example, the ‘ECM-receptor interaction’ and ‘focal adhesion’ signaling pathways were found for identified up-DEGs, while ‘chemical carcinogenesis’ and ‘tyrosine metabolism’ were found for identified down-DEGs. Figure 6(a) display the five most enriched KEGG signaling pathways for identified up-DEGs and down-DEGs, respectively.

### PPI analysis

**Figure 7.**
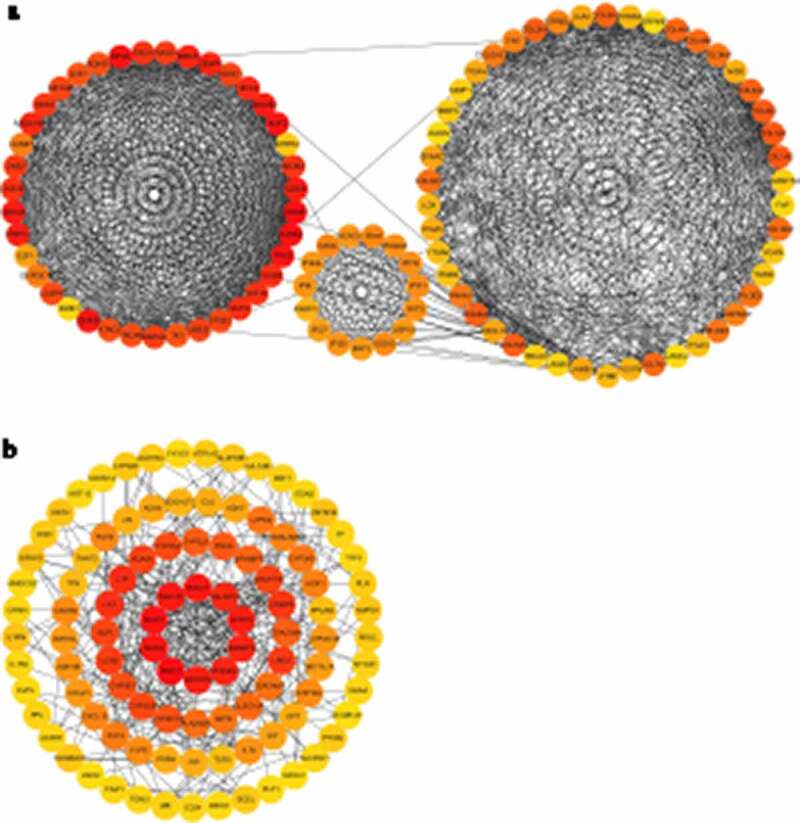
Protein-protein interaction (PPI) analysis for identified up-regulated differential expression genes (a) and identified down-regulated differential expression genes (b)

PPI analysis based on the identified up-DEGs showed that the top five hub genes related to LSCC were *CDC45, TPX2, AURKA, KIF2C,* and *NUF2* (Figure 7(a)). PPI analysis based on identified down-DEGs indicated that the top five LSCC-related hub genes were all mucin genes: *MUC1, MUC7, MUC4, MUC15,* and *MUC21* (Figure 7(b)).

### Transcription factor prediction

By using the BART tool to predict TFs related to the identified up-DEGs and down-DEGs, a total of 81 up-TFs and 101 down-TFs were screened, according to the threshold *p* < 0.01. After excluding seven duplicate TFs, 175 TFs were finally predicted for subsequent analysis.

### Identification of PRTFs and PIRTFs

Each of the eight datasets contained some of the predicted 175 TFs. Therefore, the researchers used the RNA-Seq dataset to identify PRTFs and PIRTFs, as it included most of the genes (69 of the 81 up-TFs and 96 of the 101 down-TFs) and the most complete clinical information. The 105 samples (Supplementary Material 1) with complete clinical information were included for prognosis analyses.

**Figure 8.**
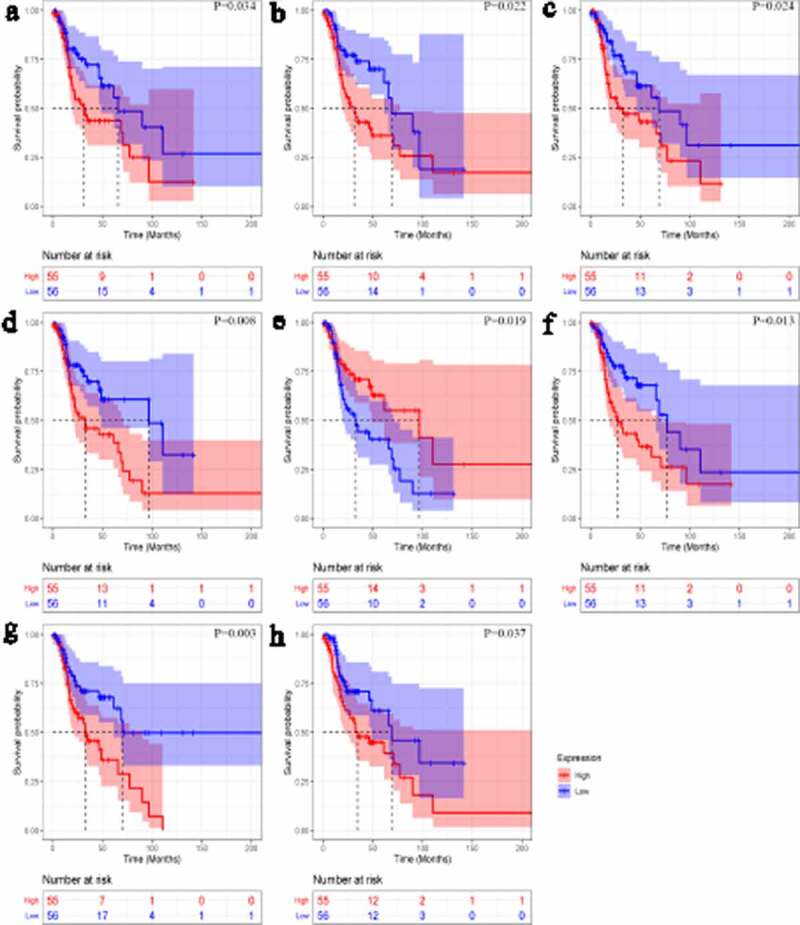
Kaplan-Meier curves of transcription factors related to laryngeal squamous cell carcinoma prognosis. *CHD4* (a); *BCAT1* (b); *FOXA2* (c); *GATA6* (d); *HNF1A* (e); *HOXB13* (f); *MAFF* (g); *TCF4* (h)

Kaplan–Meier curves showed that one up-TF (*CHD4*) and seven down-TFs (*BCAT1, FOXA2, GATA6, HNF1A, HOXB13, MAFF,* and *TCF4*) were significantly related to the overall survival rate of LSCC patients (*p* < 0.05, Figure 8). All eight TFs were identified as PRTFs.

**Figure 9.**
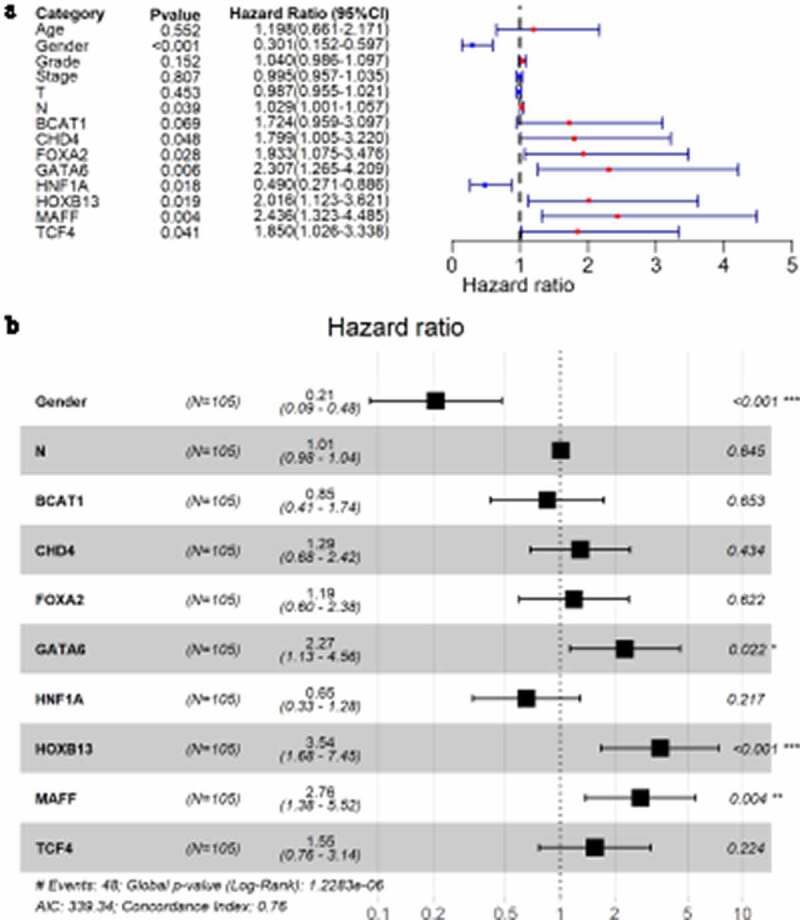
Forest plots for identifying transcription factors independently related to LSCC prognosis. A: univariate Cox analysis; B: multivariate Cox analysis. N: number of samples; * *p* < 0.05; ** *p* < 0.005; *** *p* < 0.001

Through univariate Cox analysis, LSCC patients with low *HNF1A* expression had a higher risk of death than those with high *HNF1A* expression (hazard ratio <1; Figure 9(a)), suggesting *HNF1A* may be a protective factor to LSCC prognosis. The other six (except *BCAT1*, with 95% CI of its hazard ratio including 1) PRTFs – *CHD4, FOXA2, GATA6, HOXB13, MAFF,* and *TCF4* – were risk factors to LSCC prognosis (hazard ratios >1; Figure 9(a)). Moreover, in clinical parameters, gender (male) was identified as a protective factor against LSCC prognosis. In multivariate Cox analysis, the results showed that three PRTFs – *GATA6, HOXB13* and *MAFF* – were LSCC PIRTFs and therefore risk factors (*p* < 0.05; Figure 9(b)).

### PIRTF expression in LSCC and their effects on LSCC screening

**Figure 10.**
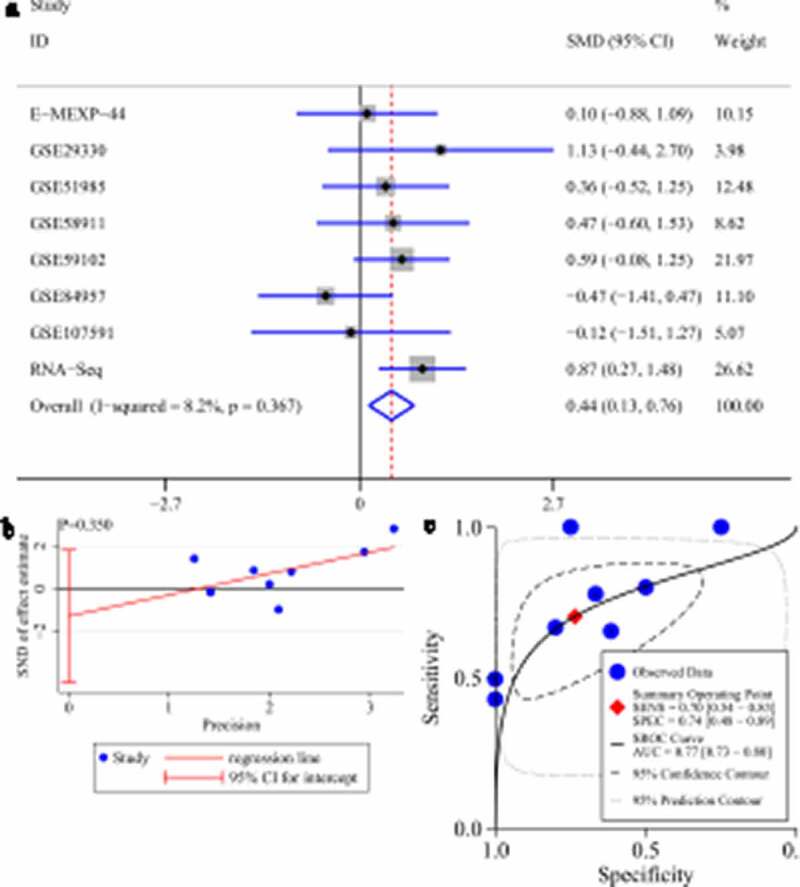
Expression of *HOXB13* and its screening effect in laryngeal squamous cell carcinoma (LSCC). A: forest plot with standard mean difference (SMD) for evaluating deferential expression of *HOXB13* between LSCC tissues and non-LSCC tissues. B: funnel plot with Egger’s test; C: summary receiver-operating curve

No difference in *GATA6* or *MAFF* expression could be seen between LSCC tissues and control tissues (95% CI contained zero; Supplementary materials 2A and 3A). No obvious publication bias was found in the SMD results (*p* value of Egger’s test >0.1; Supplementary materials 2B and 3B). Unlike *GATA6* and *MAFF, HOXB13* was significantly upregulated in LSCC tissues (SMD > 0, 95% CI [0.13–0.76]; Figure 10(a)). A *p* value of Egger’s test >0.1 indicated no obvious publication bias in the SMD results (Figure 10(b)).

Due to the differential expression of *HOXB13* between LSCC tissues and non-LSCC tissues, we performed sROC analysis based on *HOXB13* expression to detect its effect in screening LSCC. The AUC value of the sROC revealed that *HOXB13* expression made it feasible to distinguish LSCC tissues from non-LSCC tissues (Figure 10(c)), suggesting a screening effect in LSCC.

### Prediction of potential HOXB13 targets

As *HOXB13* was found to be a highly expressed PIRTF of LSCC that made LSCC screening feasible, *HOXB13* may play an important role in LSCC. To analyze the mechanism of *HOXB13* in LSCC, we explored potential *HOXB13* targets.

In Cistrome Data Browser, the high-quality and *HOXB13*-related samples (*n* = 18) revealed 17 genes – *ADD3, DNAH5, EHF, HOXA3, HOXA5, HOXA6, HOXA7, HOXA9, HOXC4, HOXC5, HOXC6, HOXC8, KLK2, LOC107984512, PHTF2, SLC36A1,* and *TMEM60* – as primary potential *HOXB13* targets with regulatory potential scores ≥2 (data not shown). Based on these eight datasets, 462 PCEGs of *HOXB13* were selected (data not shown), provided that they showed positive co-expression with *HOXB13* in more than three-eighths of the datasets. As shown above, we selected 458 identified up-DEGs. Among the 17 genes, three secondary targets – *HOXB7, HOXC8,* and *HOXC9* – were identified through the intersection of the potential targets of *HOXB13*, PCEGs of *HOXB13*, and identified up-DEGs (Supplementary material 4).

A matched sequence was found between the motif of *HOXB13* and the promoter region of *HOXB7* (Supplementary material 5). In addition, six matched sequences were identified between the motif of *HOXB13* and the promoter region *HOXC9* (Supplementary material 5), while no matched sequence was identified between the motif of *HOXB13* and the promoter region of *HOXC8*. In the three genes, some ChIP-Seq peaks of *HOXB13* were located upstream of the transcription start site of *HOXB7* (Supplementary material 6). This strongly suggests that *HOXB7* was likely a target of *HOXB13*.

As with *HOXB13, HOXB7* was upregulated in LSCC (SMD > 0; Supplementary material 7A). There was a trend that LSCC patients with high *HOXB13* and *HOXB7* expression had poorer prognoses than other LSCC patients. For example, compared with LSCC patients with low expression of both *HOXB13* and *HOXB7*, those with high *HOXB13* and *HOXB7* expression showed poorer prognosis (*p* = 4.161e-04; Supplementary material 7B).

## Discussion

Current research has revealed that multiple TFs play an important role in tumor occurrence and development, and they may be key to screening and treating tumors [41–43]. Therefore, as this study explored and identified LSCC PIRTFs, it was encouraging to discover new markers suitable for early clinical LSCC screening and treatment.

The results have indicated that identified LSCC DEGs were enriched in several tumor-related KEGG signaling pathways, suggesting that these pathways may have importance regarding LSCC. At the same time, some hub genes obtained via PPI analysis were reported in other tumor-related studies, suggesting their possible importance in LSCC development. The researchers screened eight PRTFs and three PIRTFs of LSCC via Kaplan-Meier curves and Cox analyses, which were unreported in LSCC. Moreover, *HOXB13* expression made it feasible to distinguish LSCC tissues from non-LSCC tissues through an sROC. Overall, TF *HOXB13* was found to be a potential and novel marker for LSCC screening and treatment.

Based on GO, KEGG, and PPI analyses, the researchers studied the underlying molecular mechanism of LSCC, producing results that showed some identified DEGs might play important roles in LSCC. While the top GO terms for identified down-DEGs were appreciably different, those of identified up-DEGs were relevant to the extracellular matrix. Such results indicate that extracellular matrices may be a future research direction for LSCC. The KEGG analysis showed that identified up-DEGs were enriched in ‘the ECM-receptor interaction’ and ‘focal adhesion’ signaling pathways. Identified down-DEGs were enriched in ‘chemical carcinogenesis’ and ‘tyrosine metabolism.’ Interestingly, previous research has shown these signaling pathways to be closely related to tumor development [44–46], so the current researchers speculated that changes in these pathways might be important factors in LSCC. PPI analysis of the identified up-DEGs found that *CDC45, TPX2, AURKA, KIF2C,* and *NUF2* might act as hub genes during LSCC development. Moreover, *MUC1, MUC7, MUC4, MUC15,* and *MUC21* were found to be hub genes among the identified down-DEGs. It is also interesting that some of these hub genes are closely related to tumors, such as *CDC45* [47], *TPX2* [48], *ARID1A* [49], *MUC1,* and *MUC4* [50]. Thus, some identified DEGs may be key in LSCC occurrence and development, but experimental research is required to confirm this.

**Figure:**
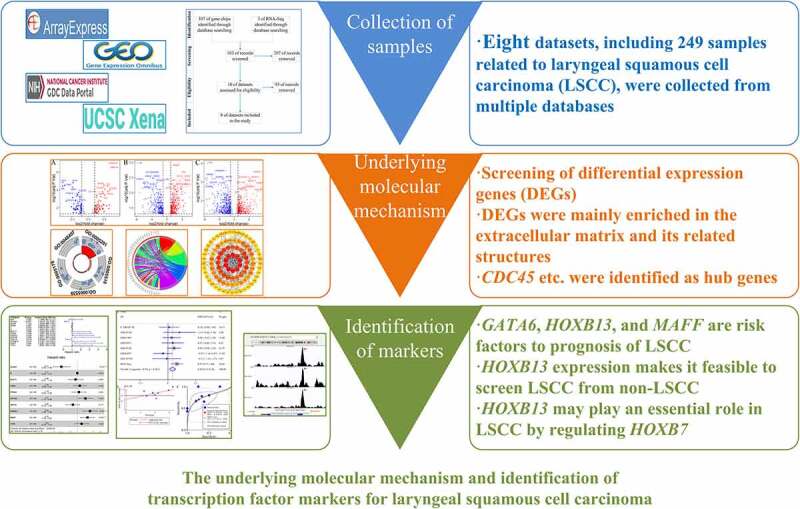

To further explore key TFs for LSCC, the researchers separately predicted the TFs related to identified up-DEGs and down-DEGs. In these TFs, PRTFs of LSCC were selected. Eight PRTFs – *BCAT1, CHD4, FOXA2, GATA6, HNF1A, HOXB13, MAFF,* and *TCF4* – had a statistically significant connection to the overall survival rate of patients with LSCC. Among them, as far as we know, in addition to *GATA6* [51], *HOXB13* [52], and *TCF4* [53], there were no reports on the remaining five PRTFs in the published LSCC research. This indicates novelty of our study to some extent. Furthermore, with independent prognostic value in LSCC, the three PIRTFs – *GATA6, HOXB13* and *MAFF* – were further analyzed in the current research.

*GATA6*, a member of the *GATA* family [54], is closely associated with the occurrence and development of multiple cancers. For instance, reduced *GATA6* expression may inhibit gastric cancer progression [55]. *GATA6* expression is related to poor prognosis in ovarian cancer [56]. However, *GATA6* may also exert an inhibitory effect on lung adenocarcinoma [57], indicating varying effects in tumors across cancer types. Previous reports concerning LSCC have also suggested that the evaluated expression of *GATA6* promotes the development of LSCC [51,58]. The current study further shows that *GATA6* is an independent risk factor for LSCC prognosis, making it a possible marker for LSCC treatment.

Another PIRTF of LSCC, *MAFF*, has also been reported in several cancers other than LSCC. For example, bladder cancer patients with high *MAFF* expression have a higher survival rate [59], suggesting a cancer suppression effect, yet it remains controversial whether *MAFF* has a tumor suppressor effect in hepatocellular carcinoma. For example, Tsuchiya et al. identified that knocking down *MAFF* expression would inhibit the invasion of hepatocellular carcinoma cells [60]; while Wu et al. believed that *MAFF* exerts a tumor suppressor effect via the circ-ITCH/miR-224-5p axis in hepatocellular carcinoma [61]. In this study, *MAFF* was identified as a risk factor for LSCC prognosis, which has not, to the best of our knowledge been reported before. This controversy over whether *MAFF* has a cancer-promoting or anti-tumor effect makes it necessary to confirm our findings with larger samples and experiments.

Although *GATA6* and *MAFF* are closely related to the prognosis of LSCC patients, there is no differential expression of either PIRTF between LSCC tissues and control tissues. High expression of *GATA6* in LSCC has been reported [51,58], but this study’s larger samples did not show *GATA6* was differentially expressed in LSCC tissues and control tissues. Similarly, no differential expression of *MAFF* was detected in LSCC. These findings suggest that, *GATA6* and *MAFF* may have screening limitations for LSCC, although they were independent risk factors of the prognosis of LSCC patients. By contrast, a PIRTF of LSCC, *HOXB13*, had not only an upregulated expression in LSCC, but also had a certain effect on LSCC screening.

*HOXB13* plays an important role in the occurrence and development of multiple tumors, including LSCC. It is located on human chromosome 17q21.32, and the TF it encodes is one of the members of the homeobox gene family [62]. High *HOXB13* expression is associated with poor survival rates for patients with glioblastoma, which may be due to the upregulation of long noncoding RNA *HOXC-AS3* transcription [63]. In malignant striated muscle tumors, *HOXB13* plays a role in promoting cancer by interfering with the differentiation of mesenchymal stem cells [64]. Previous research found *HOXB13* was upregulated in LSCC [52], which is consistent with the findings of this study via SMD calculation. Our study further revealed that high *HOXB13* expression is a risk factor for LSCC prognosis. Due to *HOXB13* expression, drug resistance can be seen in some cancers, such as breast cancer [65] and glioblastoma [63]. These findings suggest the cancer-promoting effect of *HOXB13*. However, other scholars believed that *HOXB13* may exert a tumor suppression effect given its relation to poor prognosis of right colon cancer [66] and gastric cancer [67]. In short, these reports provide a certain direction for studying the role and molecular mechanism of *HOXB13* in the above-mentioned tumors, indicating that *HOXB13* may be a potential screening and treatment marker for them. However, the mechanism of *HOXB13* in LSCC still needs to be further studied.

The current research suggests that *HOXB13* may play an essential role in LSCC by regulating *HOXB7*. We reported for the first time that *HOXB7* is a potential target of *HOXB13. HOXB7* is not only positively correlated with *HOXB13* expression, but a binding sequence between its promoter region and the *HOXB13* motif can also be seen. In addition, there is a ChIP-Seq peak of *HOXB13* in the promoter region of *HOXB7*. These strongly suggest the potential regulatory relationship of *HOXB13* to *HOXB7*. In addition, compared with LSCC patients with low *HOXB13* and *HOXB7* expression, those with high expression of both *HOXB13* and *HOXB7* showed poorer prognosis. Collectively, the current research strongly indicates that *HOXB13* may affect the occurrence and development of LSCC by regulating *HOXB7*. Nevertheless, this has not been reported before and requires confirmation by further research.

This study did have some limitations, including a relatively small sample size and limited available data, preventing the molecular mechanisms of LSCC PIRTFs from being explored in detail. In future, in vivo and in vitro experiments on LSCC PIRTFs, especially *HOXB13*, will be necessary to confirm their roles and molecular mechanisms.

## Conclusion

This research indicates that identified DEGs may play an important role in LSCC via the extracellular matrix and its related structures or pathways. *CDC45, TPX2, AURKA, KIF2C,* and *NUF2* – as well as the mucin family (*MUC1, MUC7, MUC4, MUC15,* and *MUC21*) – are likely LSCC hub genes. The authors also screened eight LSCC PRTFs for the first time – *BCAT1, CHD4, FOXA2, GATA6, HNF1A, HOXB13, MAFF* and *TCF4. HOXB13* was determined to be an essential LSCC PIRTF, and it may be used as a new marker for the early clinical screening and treatment of LSCC.

## Data Availability

All datasets used in the research can be found in ArrayExpress (https://www.ebi.ac.uk/arrayexpress/, containing the dataset of E-MEXP-44), Gene Expression Omnibus (https://www.ncbi.nlm.nih.gov/geo/, containing datasets of GSE29330, GSE51985, GSE58911, GSE59102, GSE84957, GSE107591), and the Genomic Data Download from Commons Data Portal (https://portal.gdc.cancer.gov/repository, containing an RNA-Seq dataset related to laryngeal squamous cell carcinoma) databases.

## Research highlights

1. Explored the potential molecular mechanism of LSCC via differential expression genes.

2. *GATA6, HOXB13* and *MAFF* were risk factors in LSCC prognosis.

3. *HOXB13* may be a novel marker for LSCC clinical screening and treatment.

4. *HOXB13* may play an essential role in LSCC by regulating *HOXB7*.

5. The male gender was identified as an independent prognostic factor for LSCC patients.

## Abbreviations

LC: laryngeal carcinoma; LSCC: laryngeal squamous cell carcinoma; TF: transcription factor; GEO: Gene Expression Omnibus; TCGA: the Cancer Genome Atlas; PRTF: transcription factor related to prognosis; DEG: differential expression gene; up-DEG: upregulated-differential expression gene; down-DEGs.: downregulated-differential expression gene; GO: Gene Ontology; KEGG: Kyoto Encyclopedia of Genes and Genomes; PPI: protein–protein interaction; BART: Binding Analysis for Regulation of Transcription; ChIP-Seq: chromatin immunoprecipitation sequencing; up-TF: upregulated-transcription factor; down-TF: downregulated-transcription factor; PIRTF: transcription factor independently related to prognosis; SMD: standardized mean difference; AUC: area under the curve; sROC: summary receiver-operating curve; PCEGs: positive co-expression genes; CI: confidence interval; CC: cellular components; BP: biological processes; MF: molecular functions
